# Supplementary material for: Mistakes can stabilise the dynamics of rock-paper-scissors games
Source: PLoS Comput Biol. 2021 Apr 12;17(4):e1008523. doi: 10.1371/journal.pcbi.1008523 (PMC8062094; doi:10.1371/journal.pcbi.1008523)
Supplement: S1 File — In this document we derive all results presented in the manuscript. (PDF) [file pcbi.1008523.s001.pdf]

Supporting information for the manuscript
Mistakes can stabilise the dynamics of rock-paper-scissors games

Maria Kleshnina[1,*], Sabrina S. Streipert[2], Jerzy A. Filar[3], Krishnendu Chatterjee[1]

1 Institute of Science and Technology Austria (IST Austria), Klosterneuburg, Austria,
2 Department of Mathematics and Statistics, McMaster University, Hamilton, Canada,
3 School of Mathematics and Physics, University of Queensland, Brisbane, Australia

* maria.kleshnina@ist.ac.at

Proofs for uniform execution errors

We shall recall an equivalence result on the positive affine transformation of the fitness matrix from [1].

Lemma 1. *If $\hat{R} = kR + C_c$, where $k > 0$ and C_c is some column-constant matrix, then R and $\hat{R}$ have identical evolutionary stable strategies (ESS), fixed points and their stability properties.*

Thus, we can then simplify our new game in the following manner.

Lemma 2. *If matrix S is a uniform matrix, that is, $s_{ij} = 1/n, \forall i, j = 1, \dots, n$, then the game $R(\lambda)$ (as defined in the equation (4) in the main text) is equivalent to the simplified uniform game with the fitness matrix $\hat{R}(\lambda) = R + \frac{1-\lambda}{\lambda n} RJ$, where J is a matrix of ones, R is the fitness matrix of the original game and $\lambda \neq 0$.*

Proof. Due to the fact that S is a uniform matrix we can rewrite the matrix $Q(\lambda)$ as:

$$Q(\lambda) = \frac{1-\lambda}{n} J + \lambda I = Q^T(\lambda).$$

The new fitness matrix can be rewritten as

$$R(\lambda) = \left(\frac{1-\lambda}{n}\right)^2 JRJ + \frac{\lambda(1-\lambda)}{n}\left(RJ + JR\right) + \lambda^2 R = \lambda^2 R + C_r + C_c, \tag{1}$$

where JR is a column-constant matrix with the constant element of the j-th column being $r_{*j} = \sum_i r_{ij}$. Similarly, RJ is a row-constant matrix with the constant element of the i-th row being $r_{i*} = \sum_j r_{ij}$. Finally, JRJ is a constant matrix with all elements being $\sum_j \sum_i r_{ij}$. In the above, we have set $C_c := \left(\frac{1-\lambda}{n}\right)^2 JRJ + \frac{\lambda(1-\lambda)}{n} JR$, a row-constant matrix and $C_r := \frac{\lambda(1-\lambda)}{n} RJ$ a column-constant matrix. Thus,

$$R(\lambda) = \lambda^2 \left(R + \frac{1}{\lambda^2} C_r + \frac{1}{\lambda^2} C_c\right). \tag{2}$$

Applying Lemma 1 to (2), it is sufficient to consider only the equilibria and fixed points of

$$\hat{R}(\lambda) = R + \frac{1-\lambda}{\lambda n} RJ. \tag{3}$$

□

The specific form of the fitness matrix (3) allows for the following result.

Lemma 3. *If* $\hat{R}(\lambda) = R + \frac{1-\lambda}{\lambda n} RJ$, *then* $\det(\hat{R}(\lambda)) = \frac{1}{\lambda}\det(R)$.

Proof. (i) Let us first consider the matrix $\hat{R} = R + RJ$, hence, $\hat{r}_{ij} = r_{ij} + r_{i*}$. According to [2] the determinant of such matrix can be written in the following form:

$$\det(\hat{R}) = \det(R) + \det(\tilde{\mathbf{c}}, \mathbf{c}_2 - \mathbf{c}_1, \ldots, \mathbf{c}_n - \mathbf{c}_1), \tag{4}$$

where $\mathbf{c}_j$ are columns of R and $\tilde{\mathbf{c}}$ is a column vector of row-sums of R. Note that the second term of the determinant of $\hat{R}$ is a sum of n determinants:

$$\det(\tilde{\mathbf{c}}, \mathbf{c}_2 - \mathbf{c}_1, \ldots, \mathbf{c}_n - \mathbf{c}_1) = \sum_{j=1}^{n} \det(\mathbf{c}_j, \mathbf{c}_2 - \mathbf{c}_1, \ldots, \mathbf{c}_n - \mathbf{c}_1).$$

Consider one of the determinants, in the above summation, for some j:

$$\begin{aligned}&\det(\mathbf{c}_j, \mathbf{c}_2 - \mathbf{c}_1, ..., \mathbf{c}_j - \mathbf{c}_1, ..., \mathbf{c}_n - \mathbf{c}_1) = \det(\mathbf{c}_j, \mathbf{c}_2 - \mathbf{c}_1, ..., -\mathbf{c}_1, ..., \mathbf{c}_n - \mathbf{c}_1)\\ &= -\det(-\mathbf{c}_1, \mathbf{c}_2 - \mathbf{c}_1, ..., \mathbf{c}_j, ..., \mathbf{c}_n - \mathbf{c}_1) = \det(\mathbf{c}_1, \mathbf{c}_2, ..., \mathbf{c}_j, ..., \mathbf{c}_n) = \det(R).\end{aligned}$$

Hence, from (4) it follows that $\det(\hat{R}) = (n+1)\det(R)$.

(ii) Now, let us consider the matrix $\hat{R}(\lambda) = R + \frac{\lambda(1-\lambda)}{n} RJ$. Due to the fact that the second term of the determinant of $\hat{R}(\lambda)$ in (4) is now $\det(\frac{1-\lambda}{\lambda n}\tilde{\mathbf{c}}, \mathbf{c}_2 - \mathbf{c}_1, \ldots, \mathbf{c}_n - \mathbf{c}_1)$, we obtain the required result. □

We know from [3] that there are a very special balanced bifurcations of the fixed points occurring for some values of λ. Let us first recall the definition of a balanced bifurcation parameter value and the result for the interior fixed point.

Definition 1. *We shall say that* λ^c *is a balanced bifurcation parameter value of the fixed point* $\tilde{\mathbf{x}}$ *when there exists a bifurcation of* Γ_λ *at* λ^c *and the mean fitness of the population* $\phi(\tilde{\mathbf{x}}, \lambda^c) = 0$.

Lemma 4. *[3] If* $\tilde{\mathbf{x}}$ *is an interior fixed point of* R, *that is,* $\tilde{x}_i > 0, \forall i$, *then every balanced bifurcation parameter value,* λ^c, *is also a singular point of* $\tilde{R}(\lambda)$, *that is* $\det[\tilde{R}(\lambda^c)] = 0$.

Hence, Lemmas 3-4 imply that for the case of the uniform incompetence we do not obtain any balanced bifurcation points except, perhaps, $\lambda^c = 0$. However, we may extend our conclusions and formulate the following result:

Proposition 1. *Let* $\tilde{\mathbf{x}}$ *be an interior ESS of* R. *If the starting level of incompetence,* S, *is a uniform matrix, that is,* $s_{ij} = 1/n, \forall i, j = 1, \ldots, n$ *and* R *is a row-sum-constant matrix, then* $\tilde{\mathbf{x}}$ *is an interior ESS for the incompetent game* $\hat{R}(\lambda)$, *for any* $\lambda \in [0, 1]$.

Proof. Since $\tilde{\mathbf{x}}$ is an interior ESS, by Proposition 12 in [1] it is the unique solution of the equation

$$R\tilde{\mathbf{x}} = (\tilde{\mathbf{x}}R\tilde{\mathbf{x}})\mathbf{1},$$

where $\mathbf{1}$ is a vector of ones. Therefore, R^{-1} exists and equals $\frac{1}{\det(R)}[R_{ij}]^T$, where R_{ij}'s are cofactors of R and we have

$$\tilde{x}_k = \frac{\det(R)}{\sum_{j=1}^n \sum_{i=1}^n R_{ij}} \times \frac{\sum_{j=1}^n R_{jk}}{\det(R)} = \frac{\sum_{j=1}^n R_{jk}}{\sum_{j=1}^n \sum_{i=1}^n R_{ij}}. \tag{5}$$

From Lemma 1 we can consider the simplified uniform fitness matrix $\hat{R}(\lambda) = R + \frac{1-\lambda}{\lambda n} JR$. From Lemma 2.1 of [2] we know that

$$\sum_{j=1}^{n}\sum_{i=1}^{n} R_{ij} = \sum_{j=1}^{n}\sum_{i=1}^{n} \hat{R}(\lambda)_{ij}.$$

Suppose now that $\tilde{\mathbf{x}}$ is not an interior ESS for the game with $\hat{R}(\lambda)$. Then,

$$\hat{R}(\lambda)\tilde{\mathbf{x}} \neq (\tilde{\mathbf{x}}\hat{R}(\lambda)\tilde{\mathbf{x}})\mathbf{1},$$

and, hence,

$$\tilde{x}_k \neq \tilde{x}_k(\lambda) = \frac{\sum_{j=1}^{n} \hat{R}(\lambda)_{jk}}{\sum_{j=1}^{n}\sum_{i=1}^{n} \hat{R}(\lambda)_{ij}}. \tag{6}$$

However, according to the proof of Lemma 3.3 in [2] we can rewrite the right-hand side of (6) as

$$\frac{\sum_{j=1}^{n} \hat{R}(\lambda)_{jk}}{\sum_{j=1}^{n}\sum_{i=1}^{n} \hat{R}(\lambda)_{ij}} = \frac{\sum_{j=1}^{n} R_{jk} - \gamma_k(\tilde{\mathbf{c}})}{\sum_{j=1}^{n}\sum_{i=1}^{n} R_{ij}} = \frac{\sum_{j=1}^{n} R_{jk}}{\sum_{j=1}^{n}\sum_{i=1}^{n} R_{ij}} - \frac{\gamma_k(\tilde{\mathbf{c}})}{\sum_{j=1}^{n}\sum_{i=1}^{n} R_{ij}},$$

where $\gamma_k(\tilde{\mathbf{c}}) = \det(\tilde{\mathbf{c}}, \mathbf{c}_2 - \mathbf{c}_1, ..., \mathbf{c}_{k-1} - \mathbf{c}_1, \mathbf{1}, \mathbf{c}_{k+1} - \mathbf{c}_1, ..., \mathbf{c}_n - \mathbf{c}_1)$. Note that if the matrix R is row-sum-constant, that is, $\tilde{\mathbf{c}} = \nu\mathbf{1}$ for some real ν, then $\gamma_k(\tilde{\mathbf{c}}) = 0, \forall k$. Hence, in view of (5) and (6), $\tilde{x}_k = \tilde{x}_k(\lambda)$, which is a contradiction.

Let us now check that $\tilde{\mathbf{x}}$ is also an ESS for $R(\lambda)$. For that, we need to show that $\mathbf{y}\hat{R}(\lambda)\tilde{\mathbf{x}} < \tilde{\mathbf{x}}\hat{R}(\lambda)\tilde{\mathbf{x}}, \forall \mathbf{y}$. Note that

$$\mathbf{y}\hat{R}(\lambda)\tilde{\mathbf{x}} = \mathbf{y}R\tilde{\mathbf{x}} + \frac{1-\lambda}{\lambda n}\mathbf{y}RJ\tilde{\mathbf{x}},$$

$$\tilde{\mathbf{x}}\hat{R}(\lambda)\tilde{\mathbf{x}} = \tilde{\mathbf{x}}R\tilde{\mathbf{x}} + \frac{1-\lambda}{\lambda n}\tilde{\mathbf{x}}RJ\tilde{\mathbf{x}}.$$

As $\tilde{\mathbf{x}}$ is an interior ESS for R, we have $\mathbf{y}R\tilde{\mathbf{x}} < \tilde{\mathbf{x}}R\tilde{\mathbf{x}}, \forall \mathbf{y}$. Furthermore, $R\tilde{\mathbf{x}} = (\tilde{\mathbf{x}}R\tilde{\mathbf{x}})\mathbf{1}$ and $RJ\tilde{\mathbf{x}} = R\mathbf{1} = \nu\mathbf{1}$, where ν is the sum of any row of R. Hence

$$\mathbf{y}RJ\tilde{\mathbf{x}} = \mathbf{y}R\mathbf{1} = \nu = \tilde{\mathbf{x}}RJ\tilde{\mathbf{x}}.$$

Combining the above, we obtain

$$\mathbf{y}R\tilde{\mathbf{x}} + \frac{1-\lambda}{\lambda n}\mathbf{y}RJ\tilde{\mathbf{x}} < \tilde{\mathbf{x}}R\tilde{\mathbf{x}} + \frac{1-\lambda}{\lambda n}\tilde{\mathbf{x}}RJ\tilde{\mathbf{x}}.$$

In the same manner we obtain the second condition for an ESS.

□

The above result postulates that if matrix S is uniform, then the effect of mistakes is neglected in a row-sum constant game, which induces no overall fitness advantage to any strategy. In other words, if in a row-sum constant game everyone is making the same mistakes with the same probability, then population dynamics are invariant under these mistakes. We can extend this result for the general form of the fitness matrix as follows:

Theorem 1. *Let $\mu = 1 - \lambda$ and let $\tilde{\mathbf{x}}$ be an interior ESS for R. Then, for μ sufficiently close to 0, if the starting level of incompetence, S, is a uniform matrix, that is, $s_{ij} = 1/n, \forall i, j = 1, \ldots, n$, then*

$$\tilde{\mathbf{x}}(\mu) = \frac{1}{1-\mu}\left(\tilde{\mathbf{x}} - \frac{\mu}{n}\mathbf{1}\right) \tag{7}$$

is an interior ESS for the game $\hat{R}(\lambda)$.

Proof. For the uniform game, we consider the simplified version with $\hat{R}(\lambda) = R + \frac{1-\lambda}{\lambda n} RJ$. For $\mu = 1 - \lambda$ being sufficiently close to 0, we obtain a game which possesses an ESS [4]. Then, this ESS is given by

$$\tilde{\mathbf{x}}(\lambda) = \phi(\lambda)\hat{R}^{-1}(\lambda)\mathbf{1},$$

where $\phi(\lambda)$ is a mean fitness. Hence, we need to analyse the inverse of the perturbed fitness matrix. Firstly, we note that

$$\tilde{R}^{-1}(\lambda) = \hat{R}^{-1}(1-\mu) = \left[R\left(I - \frac{\mu}{(\mu-1)n}J\right)\right]^{-1}.$$

Let $W := \frac{\mu}{(\mu-1)n}J$, then we can use the Neumann series expression to obtain the inverse of $(I - W)$ as follows

$$\hat{R}^{-1}(1-\mu) = \left(I + W + W^2 + W^3 + \ldots\right)R^{-1}.$$

Then, the ESS for the perturbed game can be expressed as

$$\tilde{\mathbf{x}}(\mu) = \phi(1-\mu)\left(I + \frac{\mu}{(\mu-1)n}J + \left(\frac{\mu}{(\mu-1)n}J\right)^2 + \left(\frac{\mu}{(\mu-1)n}J\right)^3 + \ldots\right)R^{-1}\mathbf{1}.$$

Due to the fact that $\tilde{\mathbf{x}} = \phi(1)R^{-1}\mathbf{1}$, $J^k = n^{k-1}J$ and that terms in the parenthesis are a geometric series, we can further simplify the above expression to

$$\tilde{\mathbf{x}}(\mu) = \frac{\phi(1-\mu)}{\phi(1)}\left(I + \frac{\mu}{(\mu-1)n} \times \frac{1}{1 - \frac{\mu}{\mu-1}}J\right)\tilde{\mathbf{x}} = \frac{\phi(1-\mu)}{\phi(1)}\left(I - \frac{\mu}{n}J\right)\tilde{\mathbf{x}}.$$

Note that $\phi(1) = \dfrac{det(R)}{\sum_i \sum_j R_{ij}}$ and $\phi(1-\mu) = \dfrac{det(\hat{R}(1-\mu))}{\sum_i \sum_j R_{ij}}$. Hence, by Lemma 3 the proof is complete. □

Remark 1. *Note that Proposition 1 and Theorem 1 also apply to the interior fixed point that is not an ESS.*

Proofs for the RPS game

Before stating the lemmas leading up to the main theorem, let us recall that we obtain a new game $\tilde{R}(\lambda)$ of a form

$$\tilde{R}(\lambda) = \begin{pmatrix} 0 & \tilde{r}_{12} & \tilde{r}_{13} \\ \tilde{r}_{21} & 0 & \tilde{r}_{23} \\ \tilde{r}_{31} & \tilde{r}_{32} & 0 \end{pmatrix}. \tag{8}$$

Equilibrium	Signs $\tilde{r}_{ij}$	Additional conditions
Vertex 1	$\tilde{r}_{21} < 0, \tilde{r}_{31} < 0$	–
Vertex 2	$\tilde{r}_{12} < 0, \tilde{r}_{32} < 0$	–
Vertex 3	$\tilde{r}_{13} < 0, \tilde{r}_{23} < 0$	–
Edge (1,3)	$\tilde{r}_{13} > 0, \tilde{r}_{31} > 0$	$\tilde{r}_{13}\tilde{r}_{21} + \tilde{r}_{31}\tilde{r}_{23} < \tilde{r}_{13}\tilde{r}_{31}$
Edge (1,2)	$\tilde{r}_{12} > 0, \tilde{r}_{21} > 0$	$\tilde{r}_{12}\tilde{r}_{31} + \tilde{r}_{21}\tilde{r}_{32} < \tilde{r}_{12}\tilde{r}_{21}$
Edge (2,3)	$\tilde{r}_{23} > 0, \tilde{r}_{32} > 0$	$\tilde{r}_{23}\tilde{r}_{12} + \tilde{r}_{32}\tilde{r}_{13} < \tilde{r}_{23}\tilde{r}_{32}$
Interior	–	$\tilde{r}_{12}\tilde{r}_{23}\tilde{r}_{31} + \tilde{r}_{13}\tilde{r}_{21}\tilde{r}_{32} > 0$

Table A: Stability conditions for all possible equilibria in a RPS game.

First, we recall from [1] conditions for stability of all possible equilibria in three-dimensional games whenever they exist in Table A.

That is, for example, a fixed point on the edge (1,2) is a stable point if matrix elements $\tilde{r}_{12}$ and $\tilde{r}_{21}$ are positive and the value $\tilde{r}_{12}\tilde{r}_{31} + \tilde{r}_{21}\tilde{r}_{32}$ is negative.

Lemma 5. *There exists $\epsilon > 0$ such that the interior fixed point for the perturbed RPS game* (8) *exists for $\lambda \in (1-\epsilon, 1]$.*

Proof. The interior fixed point for the RPS game (8) is

$$\tilde{x}_k = \frac{\sum_{j=1}^{3} \tilde{R}_{kj}}{\sum_{j=1}^{3}\sum_{i=1}^{3} \tilde{R}_{ji}},$$

where $\tilde{R}_{ji}$ are cofactors of the matrix $\tilde{R}(\lambda)$, that is,

$$\begin{aligned}
\tilde{x}_1(\lambda) &= \frac{\tilde{r}_{12}\tilde{r}_{23} + \tilde{r}_{13}\tilde{r}_{32} - \tilde{r}_{23}\tilde{r}_{32}}{\tilde{r}_{23}\tilde{r}_{31} - \tilde{r}_{12}\tilde{r}_{21} + \tilde{r}_{12}\tilde{r}_{23} + \tilde{r}_{12}\tilde{r}_{31} + \tilde{r}_{21}\tilde{r}_{32} - \tilde{r}_{23}\tilde{r}_{32} + \tilde{r}_{13}\tilde{r}_{21} - \tilde{r}_{13}\tilde{r}_{31} + \tilde{r}_{13}\tilde{r}_{32}} \\
\tilde{x}_2(\lambda) &= \frac{\tilde{r}_{13}\tilde{r}_{21} - \tilde{r}_{13}\tilde{r}_{31} + \tilde{r}_{23}\tilde{r}_{31}}{\tilde{r}_{23}\tilde{r}_{31} - \tilde{r}_{12}\tilde{r}_{21} + \tilde{r}_{12}\tilde{r}_{23} + \tilde{r}_{12}\tilde{r}_{31} + \tilde{r}_{21}\tilde{r}_{32} - \tilde{r}_{23}\tilde{r}_{32} + \tilde{r}_{13}\tilde{r}_{21} - \tilde{r}_{13}\tilde{r}_{31} + \tilde{r}_{13}\tilde{r}_{32}} \\
\tilde{x}_3(\lambda) &= \frac{-\tilde{r}_{12}\tilde{r}_{21} + \tilde{r}_{12}\tilde{r}_{31} + \tilde{r}_{21}\tilde{r}_{32}}{\tilde{r}_{23}\tilde{r}_{31} - \tilde{r}_{12}\tilde{r}_{21} + \tilde{r}_{12}\tilde{r}_{23} + \tilde{r}_{12}\tilde{r}_{31} + \tilde{r}_{21}\tilde{r}_{32} - \tilde{r}_{23}\tilde{r}_{32} + \tilde{r}_{13}\tilde{r}_{21} - \tilde{r}_{13}\tilde{r}_{31} + \tilde{r}_{13}\tilde{r}_{32}}
\end{aligned}$$

Note that the interior point exists only if all $\sum_{j=1}^{3} \tilde{R}_{kj}$ have the same sign. For $\lambda = 1$, numerators of $\tilde{x}_i(1)$, denoted by x_i^n are

$$x_1^n(1) = x_2^n(1) = x_3^n(1) = b^2 + a^2 + ab > 0.$$

Hence, by structure, $\tilde{x}_i(\lambda)$ is continuous. Thus, there exists $\epsilon > 0$ such that $x_i(\lambda) > 0$ for $i = 1, 2, 3$ and $\lambda \in (1-\epsilon, 1]$. □

As λ approaches 0, there can possibly be transitions such that the interior point no longer exists, while other equilibria are emerging. We aim to find conditions for other equilibria to exist, which depends on the signs of the matrix elements $\tilde{R}(\lambda)$ given by quadratic polynomials in λ. In order to analyse the

number of roots of the quadratic polynomial in $(0,1)$, we shall use the Descartes' rule of signs. For that, note that in general, every quadratic polynomial

$$F(x) = Ax^2 + Bx + C$$

can be re-written as

$$G(x) = \tilde{A}(x-1)^2 + \tilde{B}(x-1) + \tilde{C},$$

with the relations

$$A = \tilde{A}, \quad B = \tilde{B} - 2\tilde{A}, \quad C = \tilde{A} - \tilde{B} + \tilde{C}. \tag{9}$$

In Table B, we provide possible cases for the roots of the polynomial $F(x)$, where $A_s = \text{sign}(A)$.

Signs G(x)	Signs G(x)	Signs F(x)	Solutions
$\tilde{A}_s\tilde{C}_s > 0$	$\tilde{A}_s\tilde{B}_s > 0$	$A_sC_s > 0$, $A_sB_s < 0$	both roots in $(0,1)$ **or** no roots
		$A_sC_s > 0$, $A_sB_s > 0$	no positive root
		$A_sC_s < 0$	one unique root in $(0,1)$
	$\tilde{A}_s\tilde{B}_s < 0$	$A_sC_s > 0$, $A_sB_s < 0$	no roots **or** both roots in $(1,\infty)$
		$A_sC_s > 0$, $A_sB_s > 0$	no positive root
		$A_sC_s < 0$	one unique root in $(0,1)$
$\tilde{A}_s\tilde{C}_s < 0$	$\tilde{A}_s\tilde{B}_s \pm$	$A_sC_s > 0$, $A_sB_s < 0$	one unique root in $(0,1)$ (and one > 1)
		$A_sC_s > 0$, $A_sB_s > 0$	not valid
		$A_sC_s < 0$	no root in $(0,1)$ (only one root > 1)

Table B: Roots of a quadratic polynomial $F(x)$ and $G(x)$ according to the Descartes' rule of signs.

If $\tilde{A}_s\tilde{C}_s < 0$, then necessarily, there exists one positive root bigger than one. But if additionally $A_sC_s > 0$ and $A_sB_s > 0$, then this implies there is no positive root, which contradicts that there exists a positive root bigger than one. In the following result, we shall derive the polynomial form of $\tilde{r}_{ij}(\lambda)$.

Lemma 6. *Let R be an RPS game with $a_i = a > b = b_i > 0$ for $i = 1,2,3$. Every element of the corresponding fitness matrix $\tilde{R}(\lambda)$ in (8) can be expressed as*

$$\tilde{r}_{ij}(\lambda) = A_{ij}\lambda^2 + B_{ij}\lambda + C_{ij}, \tag{10}$$

where

$$\begin{aligned} A_{ij} &= -D_{ij} + C_{ij} + r_{ij}, \\ B_{ij} &= D_{ij} - 2C_{ij}, \\ C_{ij} &= (\mathbf{s}_i - \mathbf{s}_j)^T R\mathbf{s}_j, \\ D_{ij} &= r_{ij}(s_{jj} + s_{ii} - s_{ji} - s_{jk}) + r_{ji}(s_{ik} - s_{ji}). \end{aligned} \tag{11}$$

Proof. Every element of the fitness matrix $\tilde{R}(\lambda)$ has the form:

$$\tilde{r}_{ij} = (\mathbf{q}_i - \mathbf{q}_j)^T R\mathbf{q}_j. \tag{12}$$

In this case, we obtain that each $\tilde{r}_{ij}$ is at most a quadratic polynomial.
Recall that

$$Q(\lambda) = (1-\lambda)S + \lambda I,$$

then $Q(0) = S$ and we immediately obtain

$$C_{ij} = \tilde{r}_{ij}(0) = (\mathbf{s}_i - \mathbf{s}_j)^T R \mathbf{s}_j,$$

where $\mathbf{s}_j$ is the j-th row of S, mathematically understood as a column vector.

Collecting terms one can easily verify that the other coefficients of (10) are given by

$$\begin{aligned}
C_{ij} &= (\mathbf{s}_i - \mathbf{s}_j)^T R \mathbf{s}_j \\
D_{ij} &= r_{ij}(s_{jj} + s_{ii} - s_{ji} - s_{jk}) + r_{ji}(s_{ik} - s_{ji}), \\
B_{ij} &= D_{ij} - 2C_{ij}, \\
A_{ij} &= -D_{ij} + C_{ij} + r_{ij}.
\end{aligned}$$

Hence, every element $\tilde{r}_{ij}(\lambda)$ can be expressed as a polynomial with coefficients given in (11). □

Remark. If $a = b$, then $R = a \times R^0$, where

$$R^0 = \begin{bmatrix} 0 & -1 & 1 \\ 1 & 0 & -1 \\ -1 & 1 & 0 \end{bmatrix}.$$

Then, $\tilde{r}_{ij}$ can be expressed as

$$\tilde{r}_{ij}(\lambda) = a\left(\hat{A}_{ij}\lambda^2 + \hat{B}_{ij}\lambda + \hat{C}_{ij}\right),$$

with adjusted coefficients, independent of a, given by

$$\begin{aligned}
\hat{A}_{ij} &= \hat{C}_{ij} + \text{sign}(r_{ij})(1 - s_{jj} - s_{ii} + s_{jk} + s_{ik}), \\
\hat{B}_{ij} &= -2C_{ij} + \text{sign}(r_{ij})(s_{jj} + s_{ii} - s_{jk} - s_{ik}), \\
\hat{C}_{ij} &= \mathbf{s}_i^T R^0 \mathbf{s}_j.
\end{aligned} \tag{13}$$

Next, let $\delta := s_{jj} - s_{jk} + s_{ii} - s_{ik}$, then,

$$\tilde{r}_{ij}(\lambda) = a\left(C_{ij}(1-\lambda)^2 + \text{sign}(r_{ij})\lambda(\lambda(1-\delta) + \delta)\right).$$

Note that, if $\mathbf{s}_i = \mathbf{s}_j$, then $C_{ij} = 0$ and we obtain

$$\tilde{r}_{ij}(\lambda) = a * \text{sign}(r_{ij})\lambda(\lambda(1-\delta) + \delta).$$

Hence, there exists a critical value $\lambda^c = \dfrac{\delta}{\delta - 1}$ such that $\tilde{r}_{ij}(\lambda)$ changes its sign. For $\lambda^c \in (0,1)$, we require $s_{ii} < s_{ik}$.

Next, we shall obtain conditions for the roots of the polynomials $\tilde{r}_{ij}(\lambda)$ and $\tilde{r}_{ji}(\lambda)$. Let us first consider conditions when each $\tilde{r}_{ij}(\lambda)$ has exactly one root $\lambda^c \in (0,1)$, so that each $\tilde{r}_{ij}(\lambda)$ only changes its sign once in $[0,1]$.

Lemma 7. *A matrix element $\tilde{r}_{ij}(\lambda)$ changes its sign exactly once in $(0,1)$ if and only if*

$$\operatorname{sign}((\mathbf{s}_i - \mathbf{s}_j)^T R \mathbf{s}_j) = -\operatorname{sign}(r_{ij}) \tag{14}$$

Proof. By (11), we immediately have

$$\operatorname{sign}(\tilde{r}_{ij}(0)) = \operatorname{sign}(C_{ij}) \neq \operatorname{sign}(r_{ij}) = \operatorname{sign}(\tilde{r}_{ij}(1))$$

which results, since $\tilde{r}_{ij}(\lambda)$ is quadratic in λ, by the intermediate value theorem instantly in exactly one sign change of $\tilde{r}_{ij}(\lambda)$ for $\lambda \in (0,1)$.

□

We can now formulate the following result.

Theorem 2. *Suppose $r_{ij} < 0$. If*

$$(\mathbf{s}_l - \mathbf{s}_j)^T R \mathbf{s}_j < 0, \ \forall l \neq j$$

then vertex j is a stable point of the replicator dynamics with execution errors for $\lambda \in [0, \lambda^c)$, where λ^c is one of the roots in $(0,1)$ such that $\lambda^c = \min(\lambda^c_{kj}, \lambda^c_{ij})$, where $\tilde{r}_{kj}(\lambda^c_{kj}) = 0$ and $\tilde{r}_{ij}(\lambda^c_{ij}) = 0$.

Proof. First, we note that $r_{ij} < 0$, and therefore $r_{kj} > 0$. By assumption we have

$$\operatorname{sign}(\mathbf{s}_k - \mathbf{s}_j)^T R \mathbf{s}_j = -\operatorname{sign}(r_{kj}),$$

which implies, by Lemma 7, that there exists exactly one $\lambda^c \in (0,1)$ such that $\tilde{r}_{kj}(\lambda)$ changes sign at λ^c_{kj} so that $\tilde{r}_{kj}(\lambda) < 0$ for $\lambda \in [0, \lambda^c_{kj})$.

Further, if $(\mathbf{s}_i - \mathbf{s}_j)^T R \mathbf{s}_j < 0$, then $\tilde{r}_{ij}$ can either have no roots in $[0,1]$ or two roots. If there is no roots, then $\lambda^c = \lambda^c_{kj}$. Now, assume there exist two roots and λ^c_{ij} is the smallest, then $\lambda^c = \lambda^c_{ij}$. Hence, for $\lambda \in [0, \lambda^c)$, where λ^c is the minimal value of the roots of $\tilde{r}_{kj}(\lambda)$ and $\tilde{r}_{ij}(\lambda)$, the vertex j will be stable.

□

Remark. Strictly speaking, the result stated in Theorem 2 can be extended for the game with any number of strategies for $\lambda = 0$. Consider a game with n available strategies. As before, let R denote the corresponding reward matrix and S denote the corresponding matrix of the limiting distribution of behavioural mistakes. Note that both matrices are $n \times n$. Note that for $\lambda = 0$ we obtain that $R(0) = SRS$. Hence, if we reduce matrix $R(0)$ to its canonical form $\tilde{R}(0)$ as in equation (5) from the main text, then every element of such matrix will be defined as

$$\tilde{r}_{ij}(0) = (\mathbf{s}_i - \mathbf{s}_j) R \mathbf{s}_j.$$

For the vertex j to be stable, according to Table A, we require that all elements of the j-th column $\tilde{R}(0)$ have to be negative, that is,

$$(\mathbf{s}_i - \mathbf{s}_j) R \mathbf{s}_j < 0, \ \forall i \neq j.$$

If this condition holds for $\lambda = 0$, then vertex j is stable.

If the vertex j becomes stable and component x_k approaches 0, then there can exist a point on the edge (i,j). Let us consider this case in the following result.

Corollary 1. *Let $i, j, k \in \{1, 2, 3\}$ with i, j, k all different. If $r_{ij} > 0$ and*

$$sign\left((\mathbf{s}_i - \mathbf{s}_j)^T R \mathbf{s}_j\right) = -sign(r_{ij}) \tag{15}$$

and

$$sign\left((\mathbf{s}_j - \mathbf{s}_i)^T R \mathbf{s}_i\right) = sign(r_{ji}), \tag{16}$$

then there exists λ^c such that for $\lambda \in [0, \lambda^c)$ there exists an unstable fixed point on the edge $\mathbf{x}$ such that

$$x_k = 0, \qquad x_j = \frac{\tilde{r}_{ji}}{\tilde{r}_{ji} + \tilde{r}_{ij}}, \qquad x_i = \frac{\tilde{r}_{ij}}{\tilde{r}_{ji} + \tilde{r}_{ij}}.$$

Proof. Note that

$$\text{sign}(\tilde{r}_{ji}(1)) = \text{sign}(r_{ji}) = -\text{sign}(r_{ij}).$$

Then, by Lemma 7, condition (16) and Table B, $\tilde{r}_{ji}(\lambda)$ has either no roots or two roots inside $(0, 1)$. Since $\tilde{r}_{ij}(1) > 0$ and $\tilde{r}_{ji}(1) < 0$, there exists $\lambda^c > 0$ such that $\tilde{r}_{ij}(\lambda), \tilde{r}_{ji}(\lambda) < 0$ for $\lambda \in [0, \lambda^c)$.

Therefore, $\text{sign}(\tilde{r}_{ij}(\lambda)) = \text{sign}(\tilde{r}_{ji}(\lambda))$ for $\lambda \in [0, \lambda^c)$, which results in the existence of the edge point. Therefore, the 2-dimensional subgame between i and j has an interior equilibrium $\mathbf{x}$ with

$$x_k = 0, \qquad x_j = \frac{\tilde{r}_{ji}}{\tilde{r}_{ji} + \tilde{r}_{ij}}, \qquad x_i = \frac{\tilde{r}_{ij}}{\tilde{r}_{ji} + \tilde{r}_{ij}}.$$

For $\lambda \in [0, \lambda^c)$, $\dfrac{\tilde{r}_{ji}(\lambda)}{\tilde{r}_{ji}(\lambda) + \tilde{r}_{ij}(\lambda)} \in (0, 1)$ and $\dfrac{\tilde{r}_{ij}(\lambda)}{\tilde{r}_{ji}(\lambda) + \tilde{r}_{ij}(\lambda)} \in (0, 1)$. By [1], the eigenvalues of the Jacobian evaluated at this point are $-\phi(\mathbf{x}) = -\dfrac{\tilde{r}_{ji}\tilde{r}_{ij}}{\tilde{r}_{ji} + \tilde{r}_{ij}}$ with multiplicity 2 and

$$\phi_k(\mathbf{x}) = \mathbf{e}_k \tilde{R}(\lambda)\mathbf{x} - \mathbf{x}\tilde{R}(\lambda)\mathbf{x} = \tilde{r}_{ij}\tilde{r}_{ki} + \tilde{r}_{ji}\tilde{r}_{kj} - \tilde{r}_{ij}\tilde{r}_{ji}, k \neq i, j.$$

By (16), $\tilde{r}_{ji}(\lambda) < 0$ for $\lambda \in (0, 1)$ and therefore, $\text{sign}(\tilde{r}_{ij}(\lambda)) = \text{sign}(\tilde{r}_{ji}(\lambda))$ for $\lambda \in (0, \lambda^c)$, resulting in $-\phi(x) > 0$. Hence, the equilibrium is unstable for $\lambda \in (0, \lambda^c)$. □

Besides an unstable edge point, there can exist a stable point on one of the edges. In the following analysis, we consider conditions under which such point cannot exist.

Corollary 2. *For a RPS game with execution errors, if either*

1. *vertex i is stable, or*
2. *vertex j is stable, or*
3. *an interior equilibrium exists for $\lambda \in (\lambda^c, 1]$,*

then there cannot exist a stable equilibrium on the edge (i, j).

Proof. The (i,j)-edge point, $\mathbf{x}$, can be written as

$$\mathbf{x} = \eta \mathbf{e}_i + (1-\eta)\mathbf{e}_j,$$

where $\eta = \dfrac{\tilde{r}_{ij}}{\tilde{r}_{ji}+\tilde{r}_{ij}}$. By [1], its Jacobian has three eigenvalues: $-\phi(\mathbf{x})$ with multiplicity 2 and

$$\phi_k(\mathbf{x}) = \mathbf{e}_k \tilde{R}(\lambda)\mathbf{x} - \mathbf{x}\tilde{R}(\lambda)\mathbf{x},\ k \neq i,j.$$

For $\phi(\mathbf{x}) > 0$, both $\tilde{r}_{ij}$ and $\tilde{r}_{ji}$ have to be positive. This already implies that neither vertex i nor vertex j can be stable. Further,for the stability of the equilibrium we need $\phi_k(\mathbf{x}) < 0$. That is, by Proposition 24 in [1], we need

$$\tilde{r}_{ij}\tilde{r}_{ki} + \tilde{r}_{ji}\tilde{r}_{kj} < \tilde{r}_{ij}\tilde{r}_{ji}.$$

Recall that the interior equilibrium is given by

$$\tilde{x}_k = \frac{\sum_{j=1}^{3} \tilde{R}_{jk}}{\sum_{j=1}^{3}\sum_{i=1}^{3} \tilde{R}_{ij}},$$

and the transpose of a matrix of all cofactors $\tilde{R}_{ij}$ of $\tilde{R}$, denoted by $\left[\tilde{R}_{ij}\right]_{i,j=1,2,3}$, is

$$\left[\tilde{R}_{ij}\right]_{i,j=1,2,3} = \begin{pmatrix} -\tilde{r}_{23}\tilde{r}_{32} & \tilde{r}_{23}\tilde{r}_{31} & \tilde{r}_{21}\tilde{r}_{32} \\ \tilde{r}_{13}\tilde{r}_{32} & -\tilde{r}_{13}\tilde{r}_{31} & \tilde{r}_{12}\tilde{r}_{31} \\ \tilde{r}_{12}\tilde{r}_{23} & \tilde{r}_{13}\tilde{r}_{21} & -\tilde{r}_{21}\tilde{r}_{12} \end{pmatrix}.$$

Numerators of $x_i(\lambda)$, denoted by $x_i^n(\lambda)$ simplify for $\lambda = 1$

$$x_1^n(1) = x_2^n(1) = x_3^n(1) = b^2 + a^2 + ab > 0.$$

Hence, by continuity, the signs of $x_i^n(\lambda)$ will be preserved for $\lambda \in (\lambda^c, 1]$, where $\lambda^c = \max(\lambda_1^c, \lambda_2^c, \lambda_3^c)$ and λ_i^c is a root of $x_i^n(\lambda)$. Thus, the condition for the interior equilibrium to exist is

$$\tilde{r}_{ij}\tilde{r}_{ki} + \tilde{r}_{ji}\tilde{r}_{kj} > \tilde{r}_{ij}\tilde{r}_{ji}, \quad \forall i \neq j \neq k,$$

reversing the inequality required to guarantee $\phi_k(x) < 0$. Hence, as long as the interior equilibrium exists, no edge point can be stable.

□

MATLAB code

In the following we provide the MATLAB code that we used to produce Fig. 4 in the main manuscript. This code simulates a random rock-paper-scissors game and plots existence regions for an interior point and all stable vertices.

```
%% Setting the game and calculating all critical values

count = 0;
```

```
check = 0;

while check<1 && count<5000
    [V1,V2,V3, Pos1, Pos2, Pos3,R,S] = CalcRoots();

    if sum(V1.*V2.*V3.*(Pos1>0).*(Pos2>0).*(Pos3>0))>0
        check = check+1;
    end
    count = count+1;
end

if check>0
    v1 = find(V1<0.4);
    V1(v1) = 0;
    v2 = find(V2<0.4);
    V2(v2) = 0;
    v3 = find(V3<0.4);
    V3(v3) = 0;

disp(count);
La=0:0.001:1;

%% Defining existence intervals for the interior point

Pos1t = Pos1;
Pos1t(Pos1t<0)=NaN;
Pos1t(Pos1t>1)=NaN;

Pos2t = Pos2;
Pos2t(Pos2t<0)=NaN;
Pos2t(Pos2t>1)=NaN;

Pos3t = Pos3;
Pos3t(Pos3t<0)=NaN;
Pos3t(Pos3t>1)=NaN;

Intt = Pos1t+Pos2t+Pos3t;

Pos1t(isnan(Intt)==1)=NaN;
Pos2t(isnan(Intt)==1)=NaN;
Pos3t(isnan(Intt)==1)=NaN;

fIntt = find(isnan(Intt)==1);
fIntt2 = circshift(fIntt,-1);
fIntt2(1)=fIntt(1);
fini = find(fIntt2-fIntt>1);
```

```
247
248 %% Defining stability intervals for vertices
249
250 for k=1:length(V2)
251     if V2(k)==1
252     V2(k) = V2(k)+2;
253     end
254     if V3(k)==1
255     V3(k) = V3(k)+4;
256     end
257 end
258
259 VertSum = V1+V2+V3;
260
261 Ncount = diff([0 find(diff(VertSum)) numel(VertSum)]);
262 stab=zeros(1,length(Ncount));
263 sumNcount=0;
264
265 for k=1:length(Ncount)
266     sumNcount = sumNcount+Ncount(k);
267     stab(k)=VertSum(sumNcount-1);
268 end
269
270 %% Plotting the interior equilibrium
271
272     hold on
273     plot(La, Pos1t,'LineWidth', 0.8)
274     hold on
275     plot(La, Pos2t,'LineWidth', 0.8)
276     hold on
277     plot(La, Pos3t,'LineWidth', 0.8)
278     hold on
279
280     TF = isempty(fini);
281     lf = length(fIntt);
282
283     if TF==1
284         hold on
285         if fIntt(1)==1
286             plot([La(fIntt(lf)), La(fIntt(lf))], [0,1], '--r');
287         else
288             plot([La(fIntt(1)), La(fIntt(1))], [0,1], '--r');
289             plot([La(fIntt(lf)), La(fIntt(lf))], [0,1], '--r');
290         end
291     else
292         if length(fini)==1
```

```
293             hold on
294             if fIntt(1)==1
295                 plot([La(fIntt(fini)), La(fIntt(fini))], [0,1], '--r'
296                     );
297                 plot([La(fIntt(fini+1)), La(fIntt(fini+1))], [0,1], '
298                     --r');
299                 plot([La(fIntt(lf)), La(fIntt(lf))], [0,1], '--r');
300             else
301                 plot([La(fIntt(1)), La(fIntt(1))], [0,1], '--r');
302                 plot([La(fIntt(fini)), La(fIntt(fini))], [0,1], '--r'
303                     );
304                 plot([La(fIntt(fini+1)), La(fIntt(fini+1))], [0,1], '
305                     --r');
306                 plot([La(fIntt(lf)), La(fIntt(lf))], [0,1], '--r');
307             end
308         end
309     end
310
311 %% Plotting stability intervals for vertices
312
313 hold on
314 barbb=barh([La(Ncount); nan(1,length(Ncount))],'stacked','BarWidth'
315     ,0.1);
316
317 for k=1:length(stab)
318     if stab(k)==1
319         set(barbb(k),'FaceColor',[0 0.4470 0.7410]);
320     else
321         if stab(k)==3
322             set(barbb(k),'FaceColor',[0.8500 0.3250 0.0980]);
323         else
324             if stab(k)==5
325                 set(barbb(k),'FaceColor',[0.9290 0.6940 0.1250]);
326             else
327                 if stab(k)==4
328                     set(barbb(k),'FaceColor',[0.4940 0.1840 0.5560]);
329                 else
330                     if stab(k)==6
331                         set(barbb(k),'FaceColor',[0.4660 0.6740
332                             0.1880]);
333                     else
334                         if stab(k)==8
335                             set(barbb(k),'FaceColor',[0.3010 0.7450
336                                 0.9330]);
337                         else
338                             if stab(k)==9
```

```
                                    set(barbb(k),'FaceColor',[0.6350
                                        0.0780  0.1840]);
                                else
                                    if stab(k)==0
                                     set(barbb(k),'FaceColor',[1  1  1])
                                         ;
                                    end
                                end
                            end
                        end
                    end
                end
            end
        end
    end
end

%% General plotting settings

    legend('X1', 'X2', 'X3','Position',[0.7  0.2  0.1  0.1]);
    text(0.75,0.6,{'\color[rgb]{0 0.4470 0.7410} V1', '\color[rgb
        ]{0.8500 0.3250 0.0980} V2','\color[rgb]{0.9290 0.6940 0.1250}
         V3',...
         '\color[rgb]{0.4940 0.1840 0.5560} V1-V2','\color[rgb]{0.4660
             0.6740 0.1880} V1-V3','\color[rgb]{0.3010 0.7450 0.9330}
            V2-V3',...
         '\color[rgb]{0.6350 0.0780 0.1840} V1-V2-V3'});
    ylim([0,1.1]);
    xlim([0,1]);
    grid on

end

function[V1,V2,V3, Pos1, Pos2, Pos3,R,S] = CalcRoots()
%% Parameters set and preparations

    a = rand*10; %choose a at random
    b = rand*a; % make sure b<a

    R = [[0, -a, b];[b,0,-a];[-a,b,0]];  %set up  R
    S1 = rand(3,3);  %choose S at random
    S = S1./(sum(S1').*ones(3,3))';  %normalize such that row-sum is
        1

    La=0:0.001:1;  %pick lambda values

    V1 = [];
```

```
384       V2 = [];
385       V3 = [];
386       E12 = [];
387       E13 = [];
388       E23 = [];
389       Int = [];
390       Pos1 = [];
391       Pos2 = [];
392       Pos3 = [];
393
394   %% Calculating all critical values
395
396       for la=1:length(La)
397           lambda = La(la);
398           Q = (1-lambda)*S+lambda*eye(3,3);    %set up Q
399           RL = mtimes(mtimes(Q, R), Q');    %set up R(lambda)=QRQ^T
400           RLT = RL-(diag(RL).*ones(3,3))'; %set up tilde(R)(lambda) = R
401               (lambda)-DR(lambda)
402
403           Check = (RLT<0);  %check stabilities
404           V1 = [V1, Check(2,1)*Check(3,1)];
405           V2 = [V2, Check(1,2)*Check(3,2)];
406           V3 = [V3, Check(1,3)*Check(2,3)];
407
408           E13 = [E13, (1-Check(1,3))*(1-Check(3,1))*(RLT(1,3)*RLT(2,1)+
409               RLT(3,1)*RLT(2,3)<RLT(3,1)*RLT(1,3) )];
410           E12 = [E12, (1-Check(1,2))*(1-Check(2,1))*(RLT(1,2)*RLT(3,1)+
411               RLT(2,1)*RLT(3,2)<RLT(1,2)*RLT(2,1) )];
412           E23 = [E23, (1-Check(2,3))*(1-Check(3,2))*(RLT(2,3)*RLT(1,2)+
413               RLT(3,2)*RLT(1,3)<RLT(2,3)*RLT(3,2) )];
414
415           Int = [Int, (RLT(1,2)*RLT(2,3)*RLT(3,1)+RLT(1,3)*RLT(2,1)*RLT
416               (3,2)>0)];
417
418           Num1 = RLT(1,2)*RLT(2,3)+RLT(1,3)*RLT(3,2)-RLT(2,3)*RLT(3,2);
419           Num2 = RLT(1,3)*RLT(2,1)-RLT(1,3)*RLT(3,1)+RLT(2,3)*RLT(3,1);
420           Num3 = -RLT(1,2)*RLT(2,1)+RLT(1,2)*RLT(3,1)+RLT(2,1)*RLT(3,2)
421               ;
422           Denom = Num1+Num2+Num3;
423
424           Pos1 = [Pos1, Num1/Denom];
425           Pos2 = [Pos2, Num2/Denom];
426           Pos3 = [Pos3, Num3/Denom];
427
428
429       end
```

end

References

[1] I.M. Bomze. Non-cooperative two-person games in biology: A classification. International Journal of Game Theory, 15:31–57, 1986.

[2] J. A. Filar. Semi-antagonistic equilibrium points and action costs. Cahiers du C.E.R.O., 25(3-4):227–239, 1984.

[3] M. Kleshnina, J. A. Filar, V. Ejov, and J. C. McKerral. Evolutionary games under incompetence. Journal of mathematical biology, 77(3):627–646, 2018.

[4] I.M. Bomze. Lotka-Voltera equation and replicator dynamics: a two-dimensional classification. Biological Cybernetics, 48:201–211, 1983.
