# Supplementary material for: Mistakes can stabilise the dynamics of rock-paper-scissors games
Source: PLoS Comput Biol. 2021 Apr 12;17(4):e1008523. doi: 10.1371/journal.pcbi.1008523 (PMC8062094; doi:10.1371/journal.pcbi.1008523)
Supplement: Attachment — Submitted filename: List of changes in the main text.pdf [file pcbi.1008523.s002.pdf]

List of changes in the main text of the manuscript

“Mistakes can stabilize the dynamics of rock-paper-scissors games”

by Maria Kleshnina, Sabrina Streipert, Jerzy Filar and Krishnendu Chatterjee

We would like to thank the two anonymous reviewers for their feedback on this manuscript. Please find below the list of changes made to the manuscript:

1. Included more recent references to the introduction (lines 36,40-46 and 57);
2. Motivated why we chose the unstable version of the rock-paper-scissors game (lines 99-102);
3. Commented on why it is sufficient to consider a simplified fitness matrix (lines 129-130);
4. Added comments about time-dependency of λ (lines 164-166 and 171-173);
5. Added comments to the captions of Figures 2, 3 and 4;
6. Added sections Acknowledgments and Data and Code Availability to the main body;
7. Added MATLAB code section to the Supporting Information file.
